# Differential Type-I Interferon Response in Buffy Coat Transcriptome of Individuals Infected with SARS-CoV-2 Gamma and Delta Variants

**DOI:** 10.3390/ijms241713146

**Published:** 2023-08-24

**Authors:** Guilherme C. da Fonseca, Liliane T. F. Cavalcante, Otávio J. Brustolini, Paula M. Luz, Debora C. Pires, Emilia M. Jalil, Eduardo M. Peixoto, Beatriz Grinsztejn, Valdilea G. Veloso, Sandro Nazer, Carlos A. M. Costa, Daniel A. M. Villela, Guilherme T. Goedert, Cleber V. B. D. Santos, Nadia C. P. Rodrigues, Fernando do Couto Motta, Marilda Mendonça Siqueira, Lara E. Coelho, Claudio J. Struchiner, Ana Tereza R. Vasconcelos

**Affiliations:** 1 Laboratório de Bioinformática, Laboratório Nacional de Computação Científica, Petrópolis, Rio de Janeiro 25651-076, Brazil; guicf13@gmail.com (G.C.d.F.); liliane.tavaresdefaria@gmail.com (L.T.F.C.); tavinbio@lncc.br (O.J.B.); 2 Instituto Nacional de Infectologia Evandro Chagas, FIOCRUZ, Rio de Janeiro 21040-360, Brazil; luzpaulamendes@gmail.com (P.M.L.); deboracastanheira1@gmail.com (D.C.P.); emilia.jalil@gmail.com (E.M.J.); teachereduardo@outlook.com (E.M.P.); beatriz.grinsztejn@gmail.com (B.G.); valdilea.veloso@gmail.com (V.G.V.); sandro.nazer@ini.fiocruz.br (S.N.); laraesteves@gmail.com (L.E.C.); 3 Escola Nacional de Saúde Pública, FIOCRUZ, Rio de Janeiro 21041-210, Brazil; carloscosta@ensp.fiocruz.br (C.A.M.C.); nadiacristinapr@gmail.com (N.C.P.R.); 4 Programa de Computação Científica (PROCC), FIOCRUZ, Rio de Janeiro 21040-900, Brazil; daniel.villela@fiocruz.br; 5 Escola de Matemática Aplicada (EMAp), Fundação Getúlio Vargas, Rio de Janeiro 22250-900, Brazil; guilherme.goedert@fgv.br; 6 Instituto de Medicina Social Hesio Cordeiro (IMS), Universidade do Estado do Rio de Janeiro, Rio de Janeiro 20550-013, Brazil; cleber.vini@yahoo.com.br; 7 Instituto Oswaldo Cruz, FIOCRUZ, Rio de Janeiro 21040-360, Brazil; fcm@ioc.fiocruz.br (F.d.C.M.); mmsiq@ioc.fiocruz.br (M.M.S.)

**Keywords:** SARS-CoV-2 variants, Delta, Gamma, innate immune system, type-I interferon response, viral evolution

## Abstract

The innate immune system is the first line of defense against pathogens such as the acute respiratory syndrome coronavirus 2 (SARS-CoV-2). The type I-interferon (IFN) response activation during the initial steps of infection is essential to prevent viral replication and tissue damage. SARS-CoV and SARS-CoV-2 can inhibit this activation, and individuals with a dysregulated IFN-I response are more likely to develop severe disease. Several mutations in different variants of SARS-CoV-2 have shown the potential to interfere with the immune system. Here, we evaluated the buffy coat transcriptome of individuals infected with Gamma or Delta variants of SARS-CoV-2. The Delta transcriptome presents more genes enriched in the innate immune response and Gamma in the adaptive immune response. Interactome and enriched promoter analysis showed that Delta could activate the INF-I response more effectively than Gamma. Two mutations in the N protein and one in the nsp6 protein found exclusively in Gamma have already been described as inhibitors of the interferon response pathway. This indicates that the Gamma variant evolved to evade the IFN-I response. Accordingly, in this work, we showed one of the mechanisms that variants of SARS-CoV-2 can use to avoid or interfere with the host Immune system.

## 1. Introduction

The innate immune system is the first activation point of defense against viruses and other pathogens. It involves the perception of foreign material and the activation of the interferon (IFN) cascade. One of the phases of this activation is the recognition of virus-derived molecular signatures, such as viral nucleic acids in the cytoplasm [1]. This activation is important for initiating the response against the microorganism. Therefore, the success of a new pathogen depends on its ability to block or disrupt the innate immune response in its host [2]. The coronaviridae family is a large group of animal viruses that have successfully emerged in the human population in recent decades, causing outbreaks and diseases [3]. The most recent human coronaviruses were SARS-CoV, which emerged in 2003; HCoV NL63 in 2004; HKU1 in 2005; MERS-CoV in 2012; and SARS-CoV-2 in late; 2019,which caused the COVID-19 global pandemic [3,4,5,6,7]. Studies before the COVID-19 pandemic showed that SARS-CoV could inhibit the activation of IFN-I promoter in the early stage of infection, and SARS-CoV-2 showed a similar effect in a recent study [8,9,10]. The expression of IFN-I in the initial phase of viral infection is crucial for inhibiting viral replication, preventing tissue damage, and promoting innate and adaptive immune responses [11]. Prior work showed that individuals with a deregulated antiviral response promoted by interferon are more likely to develop severe COVID-19 symptoms [11]. Early inhibition of IFN expression by the virus may benefit viral replication, leading to delayed production of inflammatory cytokines and causing more severe clinical symptoms [12].

Since SARS-CoV-2 undergoes constant genetic mutations that may confer an evolutionary advantage, it is important to monitor infected individuals for possible changes in the initial stages of the immune response to the virus by different viral genetic variants [13]. The Gamma variant, also known as P.1 variant, was identified in November 2020 in Brazil, driving the second wave of COVID-19 in Brazil [14,15]. The Delta variant was identified in December 2020 in India, and was responsible for the deadly second wave of COVID-19 in April 2021 [16]. When the Delta variant arrived in Brazil, it became dominant and replaced Gamma as the most prevalent variant until July 2021 [17,18]. Here, we aimed to evaluate the buffy coat transcriptional profile of individuals infected with SARS-CoV-2 Gamma and Delta variants.

## 2. Results

### 2.1. Delta and Gamma Groups’ Differentially Expressed Genes

**Figure 1:**
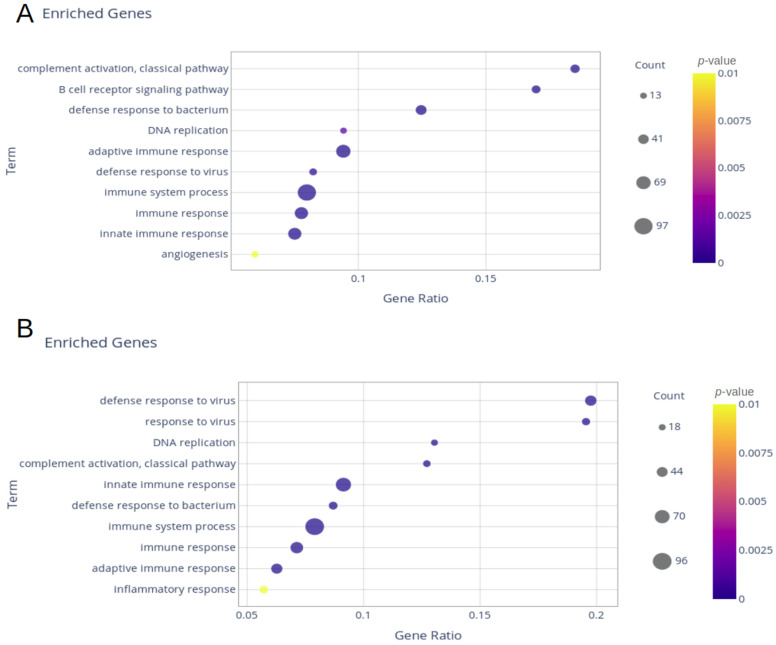
Dot blot of Gene Ontology (GO) enrichment of all differentially expressed genes (DEGs): (**A**) Gamma group; (**B**) Delta group.

Blood samples from seven individuals infected with the Delta variant, nine infected with the Gamma variant, and eight healthy controls were used in this work. The transcriptome from the Delta group showed 442 upregulated and 240 downregulated differentially expressed genes (DEGs) compared with healthy controls. Samples from the Gamma group showed 653 DEGs (370 upregulated and 283 downregulated). Gene Ontology (GO) and reactome enrichments revealed immune-systems-related pathways among the 10 terms with higher gene ratio in both groups: defense response to the virus, innate immune response, immune system process, immune response, adaptive immune response, and defense response to the bacterium (Figure 1A,B).

**Figure 2:**
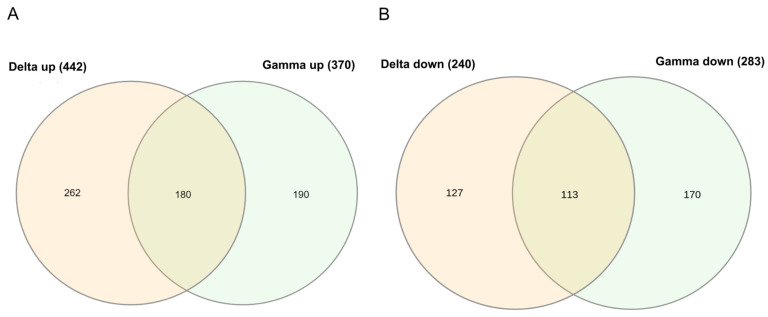
Venn diagrams: (**A**) upregulated and (**B**) downregulated shared and exclusively DEGs between Gamma and Delta transcriptomes. Only results for FDR *p* < 0.05 are displayed.

Considering the immune system term, which showed the highest value of the immune-related biological processes in GO, a similar number of genes was found (Delta = 96 and Gamma = 97, Supplementary Tables S1 and S2). The angiogenesis biological process was only enriched in the Gamma group, while the inflammatory response biological process was only enriched in the Delta group. To investigate the unique DEGs of each variant, a Venn diagram was used (Figure 2A,B). There were 293 shared deregulated genes, 180 upregulated, and 113 downregulated. The Delta group presented more exclusive upregulated genes (*n* = 262), and Gamma presented more downregulated genes (*n* = 170, Supplementary Tables S3–S6).

**Figure 3:**
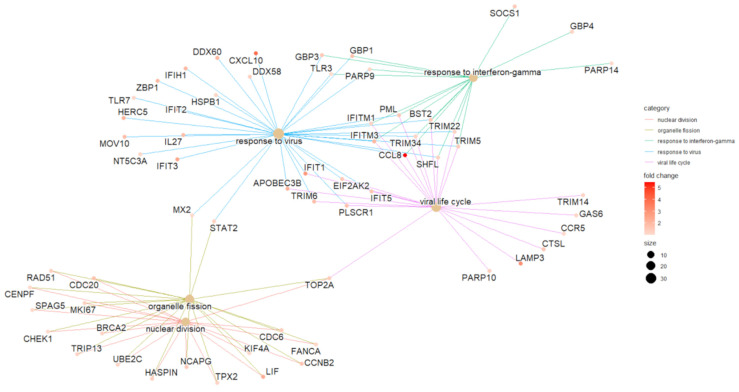
Reactome enrichment cnetplot of exclusively upregulated DEGs of Delta-infected group.

Using the exclusive DEGs of each variant group, a reactome enrichment analysis was performed. Only the upregulated genes exclusive to Delta could produce five enriched pathways: response to the virus, response to interferon-gamma, viral life cycle, organelle fission, and nuclear division processes. The genes linked to each enriched pathway are plotted in Figure 3.

Additionally, promoter motifs enrichment analysis of all deregulated genes revealed interferon-responsive genes in the Delta group, including *IRF7*, *IRF2*, and *IRF1* (Table 1). Unlike Delta, genes exclusively differentially expressed in Gamma could not produce enriched pathways in the reactome or enriched promoter regions related to the interferon response.

### 2.2. Interactome Analysis

**Figure 4:**
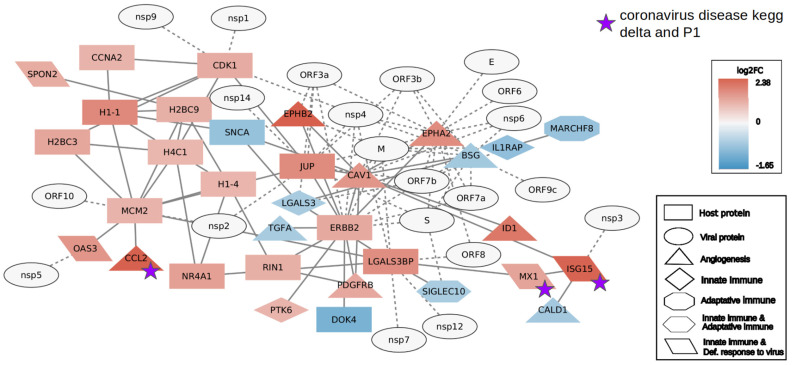
Gamma interactome of the top 20 most connected differentially expressed genes (DEGs), defense response to virus genes, angiogenesis genes, adaptive and innate immune genes, and SARS-CoV-2 proteins. Genes with a purple star are genes that are part of the SARS-CoV-2 disease pathway and are found in Delta and Gamma DEGs.

**Figure 5:**
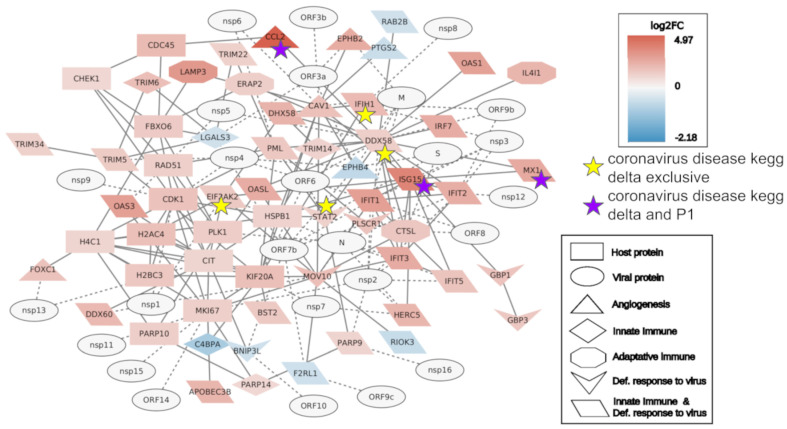
Delta interactome of the top 20 most connected differentially expressed genes (DEGs), defense response to virus genes, angiogenesis genes, adaptive and innate immune genes, and SARS-CoV-2 proteins. Genes with a purple star are genes that are part of the SARS-CoV-2 disease pathway and are found in Delta and Gamma DEGs. Genes with a yellow star are genes that are part of the SARS-CoV-2 disease pathway and are found only in Delta DEGs.

To better understand the impact on the gene regulation of each variant, interactome analysis in the Gamma and Delta groups was performed. Protein genes with a large number of connections among all deregulated protein genes can be considered hubs in cell gene expression. To focus on the host immune response, the top 20 most connected DEGs were added to the genes classified as a defense response to the virus, adaptive, and innate immune genes. Angiogenesis genes were also included in the network, as this category was exclusively enriched in the Gamma group. Moreover, SARS-CoV-2 proteins were added to evaluate their potential impact on the gene regulation of the host. The Delta group showed a larger quantity of DEGs in the network (77) with 44 upregulated, 8 downregulated, and 25 SARS-CoV-2 genes, while the Gamma network presented 24 upregulated, 9 downregulated, and 21 SARS-CoV-2 genes (Figure 4 and Figure 5).

The Delta group interactome presented 31 genes of the innate immune system, with 5 exclusive of this pathway, while the Gamma group presented only 8 with 3 exclusive of the pathway. The two networks showed only six genes involved in the adaptive immune response: four in Delta, all upregulated; and two in Gamma, both downregulated. Regarding the defense response to virus genes, Delta presented 24 genes and 6 exclusives, while Gamma presented only 4 genes related to innate immune response. At last, Gamma showed nine genes related to angiogenesis, three of which were downregulated, and Delta showed six in total, two of which were downregulated. The poly (ADP-ribose) polymerase (PARP) family was well represented in the Delta interactome (*PARP9*, *10*, *11*, *12*, and *14*) while absent in all Gamma DEGs.

We also checked which of the genes in the networks were also present in the KEGG SARS-CoV-2 pathway (Supplementary Figure S1). Delta presented seven genes, five related to the innate immune system and defense response (*EIF2AK2*, *ISG15*, *MX1*, *IFIH1*, and *DDX58*), one to angiogenesis (*CCL2*), and one exclusive to the defense response to the virus (*STAT2*). Gamma presented only three (*CCL2*, *MX1*, and *ISG15*), which were also present in the Delta interactome. Considering the SARS-CoV-2 proteins, the N protein, pivotal in the pathogenesis and evasion of the immune system, is linked to eight proteins in the Delta network but was not presented in the Gamma network.

### 2.3. Variant Mutations of Interest

To further analyze the differences between the two variants and their impact on the transcriptome, we looked for SNPs and indels. First, we selected only the mutations found in at least six participants from each group. Then, we searched for mutations that could interfere with the IFN response already described in the literature [19]. Four mutations were identified in the variants with those features: one deletion in the nsp6 of Gamma, two amino acid changes in the N protein of Gamma, and one in Delta (Table 2). Finally, we identified the proteins of SARS-CoV-2 with virulence functions that interfere with host proteins [20]. Mutations in those proteins were selected if the associated host proteins were also deregulated in our transcriptome analysis (Table 2).

## 3. Discussion

In this work, we studied a group of 24 individuals, including infected participants and healthy controls. Though this is a low number of cases to extract demographic-based conclusions, there are still some important clues to assess how differently the variants act in the immune system of the host.

The innate immune system plays an important role as the first line of defense against COVID-19, considering that most of the population did not have an extended cross-reaction to other coronaviruses [13], and there were no specific treatments or vaccines at the beginning of the pandemic.

**Figure 6:**
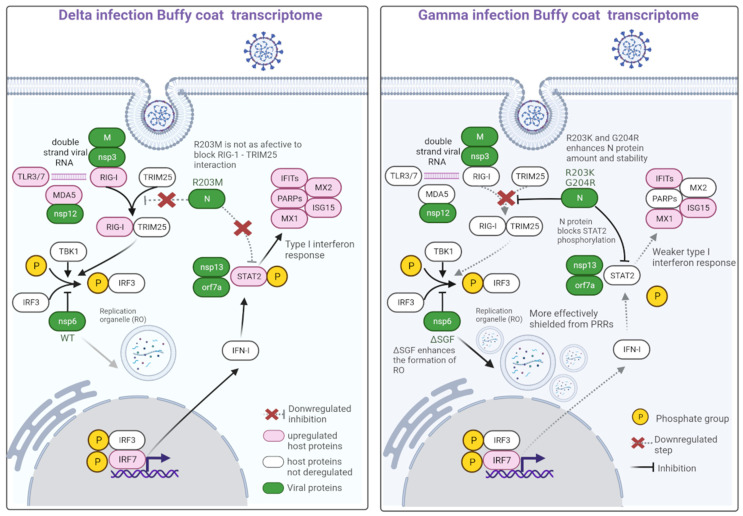
Type-I interferon response triggered by Delta and Gamma. On the left is a schematic showing the genes differentially expressed in the transcriptome of individuals infected with the Delta variant and the interaction with viral proteins and mutations that affect the interferon response. On the right is the same scheme with the transcriptome of people infected with Gamma. PRRs—pattern recognition receptors. Created with biorender.com (accessed on 7 July 2023).

After the virus entry in the cell via endocytosis, one of the first steps of the innate immune activity is the recognition of the viral particles via pattern recognition receptors (PRR), which include Toll-like receptors (TLR) in the endosomes and RIG-I-like receptors in the cytoplasm [21]. Upon activation, these PRRs induce a strong IFN response by downstream signaling cascades [13]. In the Delta transcriptome, TLR3/7 and DDX58 (RIG-I) were upregulated, which indicates that the cell was able to recognize the Delta variant and start the activation of the type-I interferon response cascade [22,23]. One of the subsequent steps of this cascade is the phosphorylation of IRF3 and IRF7, which are key regulators of the expression of type-I IFN and IFN-stimulated genes (ISGs) (Figure 6). Type-I interferon signaling also leads to phosphorylation of STAT1 and STAT2, which further interacts with IRF9 to form the ISGF3 transcription factor complex, also activating ISGs, which completes the interferon response [24].

Most of the aforementioned genes were found upregulated in the Delta transcriptome. In this transcriptome, *IRF7* was the promoter region most present in all deregulated genes (Table 1). Furthermore, *STAT2* and several ISGs, such as IFITs, IFITMs, TRIMs, and *MX2*, were exclusively upregulated in the Delta transcriptome (Supplementary Table S3). These ISGs are essential for the antiviral response in the cell. IFITs inhibit the replication of the virus through multiple mechanisms such as translation inhibition, recognition of the lack of 2′-O methylation and uncapped 5′ RNA, and binding to viral proteins or viral RNA [25]. IFITMs are transmembrane proteins that help restrict the infection of the enveloped virus by making cells refractory to steps in the viral infection cycle that precedes viral fusion [25]. Although the overexpression of IFITMs blocks SARS-CoV-2 infection, it has been demonstrated that they can also act as cofactors for efficient SARS-CoV-2 entry and replication in lung cells, cardiomyocytes, and gut organoids [26]. TRIM proteins exhibit a wide range of activities, including E3 ubiquitin ligase, SUMOylation, and ISGylation [24]. TRIM5 can bind to HIV-1 viral capsid proteins, leading to uncoating and exposure of the nucleoprotein complex, thus inhibiting the first stages of infection [24]. TRIM22 can inhibit viral replication through multiple mechanisms [24]. MXs proteins are active mainly against RNA viruses via the inhibition of the early steps of viral replication [27,28]. In influenza, MX1 can generate a protective antiviral response by controlling the expression of key molecules associated with virus lethality [29]. Mx2 is localized to the nuclear envelope and blocks the nuclear import of viral cDNAs of HIV-1 [30].

The Gamma transcriptome presents fewer DEGs associated with the IFN response, including *TLR6*, *IFITM10*, *MX1*, and *IRF7*, albeit, with a lower expression (1.33 log2Fc in Gamma and 2.46 in Delta) and no promoter regions enriched. Furthermore, *TRIM9*, *TRIM58*, and *TRIM61* are downregulated (Supplementary Table S6). Only genes exclusively upregulated by Delta could be enriched in response to the IFN-gamma category, which includes most of the genes mentioned above (Figure 3). The lack of the differential expression of IFN-response genes could indicate that Gamma can inhibit the antiviral IFN response, at least to a greater extent than Delta. To corroborate this finding, three mutations that likely confer resistance to the IFN response [19] were found exclusively in the viruses sequenced in Gamma participants (Table 2 and Figure 6).

Through RNA-seq analysis, it is possible to study the changes in the gene expression that usually correlates to changes in the protein levels. Assuming this, we looked for SARS-CoV-2 protein mutations that might affect the host immune system. The R203K/G204R mutation in the N gene was found exclusively in the viruses sequenced from Gamma-infected individuals in this study, which increased the expression of the nucleocapsid transcript [19]. These mutations increased the interaction with GSK3A kinase and allowed a hyperphosphorylation of the adjacent serine site [31]. The phosphorylation of the N protein by GSK3A is critical for the regulation of the virus life cycle and the production of viral particles [32]. These findings indicate that the N protein could be more abundant in Gamma-infected individuals. The SARS-CoV-2 nucleocapsid protein can antagonize type-I IFN signaling by interfering with the interaction of STAT1 with JAK1 and STAT2 with TYK2, thus inhibiting phosphorylation and, by interacting directly with STAT proteins, suppressing their nuclear translocations [33]. Furthermore, the nucleocapsid protein can bind TRIM25, blocking its activation by RIG-I [34]. Hence, the R203K/G204R mutation decreases the expression of ISG transcripts by inhibiting two of the major type-I IFN signaling pathways, corroborating the data from our transcriptome analysis (Figure 6).

The deletion ΔSGF in the NSP6 protein is missing in the Delta variant but present in all other variants of concern including Gamma [35]. This deletion enhances the formation of zippered endoplasmic reticulum (ER) membrane compartments that connect double-membrane vesicles to the ER [36]. This ER-derived membranous structure is the primary site of viral genome replication [36]. It is speculated that the higher zippered activity of NSP6ΔSGF could establish a replication organelle that is more functional and better shielded from innate immunity [36]. The lower availability of the viral RNA molecules during replication might negatively impact one of the first steps of innate immune activation, which is the recognition of the virus by PRRs, corroborating our data, as *TLR3*, *TLR7*, and *RIG-I* are only upregulated in the Delta transcriptome (Figure 6).

Social distancing may have driven viral evolution and changed the transmission dynamics of the virus. A larger susceptible population maintained for longer periods of time is expected under social distancing policies, slowing the spread of infection [37]. Disease severity, leading to higher hospitalization and death rates, restricts viral transmission. Viral evolution is also driven by the immunity conferred by vaccines. In this context, natural or vaccine-induced immunity may enhance viral transmission by reducing disease severity [38]. In Brazil, the Delta variant replaced Gamma with an estimated transmission advantage of 33% without increasing the number of cases or deaths reported [39]. However, this may have been due to increased immunity either due to previous infection or higher vaccine coverage. These data corroborate our analysis, as the Delta variant activates a higher number of innate immune response genes than the Gamma variant. The expression of INF-I response genes promotes NK cell activation. A higher frequency of these cells is associated with asymptomatic SARS-CoV-2 infection [40,41]. We speculate that the previous mechanisms may have contributed to the higher transmission rate of the Delta variant as more than half of all transmission of SARS-CoV-2 is accounted for by asymptomatic individuals [42]. In the most recent Omicron variant, R203K/G204R mutations in the N protein and ΔSGF deletion in NSP6 are present, as in the Gamma variant, which indicates that the worldwide spreading of Omicron might have occurred via different mechanisms in comparison with Delta.

This work demonstrates one of the mechanisms by which SARS-CoV-2 variants can improve infection and virulence by interfering with host proteins. We show that these two variants differ in their ability to inhibit the interferon response in individuals infected with SARS-CoV-2. This is supported by gene expression and interactome analyses, which corroborate the findings of previous researchers that studied the interferon response in tissue culture samples and animal models [43].

## 4. Materials and Methods

### 4.1. Study Participants and Sample Collection

The samples were collected at households in the Manguinhos neighborhood in Rio de Janeiro, Brazil. Recruitment was based on suspected SARS-CoV-2 infection, defined as either a confirmed diagnosis with PCR or presence of clinical signs in at least one household member, between April 2021 and October 2021. All household members were invited to participate in the study. All household members had blood samples and nasal swabs collected by trained laboratory technicians. Between 2.5 mL and 10 mL of blood was collected in an anticoagulant tube. The buffy coat fraction was then separated via centrifugation at 1500–2000× *g* for 15 min and then stored in a ratio of 1:2 of RNA at −80 °C until RNA extraction. Samples were selected based on the following criteria: (a) cycle threshold (Ct) < 35 for quantitative PCR for SARS-CoV-2 targets, (b) from participants with a nonreactive serological test but positive in the same test two weeks later, and (c) having the variant sequenced or from someone in the same household. We used these criteria to ensure that the samples were collected during a similar period of infection. 

### 4.2. Transcriptome Sample Processing and Sequencing

The total RNA of the buffy coat samples was extracted to carry out the transcriptome analysis with a commercial RiboPure^TM^ RNA Purification Kit (Thermo Fisher Scientific, San Jose, CA, USA), according to the manufacturer’s instructions. RNA quality and quantity were measured using a NanoPhotometer spectrophotometer, a Qubit RNA Assay Kit in Qubit 2.0 Fluorometer (Life Technologies, Carlsbad, CA, USA), and TapeStation (Agilent Technologies, Santa Clara, CA, USA). According to the manufacturer’s protocol, an average amount of 0.3 μg total RNA was used to build the libraries using a TruSeq Stranded Total RNA Library Prep Gold Kit (Illumina, San Diego, CA, USA). The Illumina NextSeq platform was used to generate 75 bp paired-end reads.

### 4.3. SARS-CoV-2 Sequencing

The whole-genome sequencing of the samples was performed using the COVIDSeq Illumina test protocol adapted by the Fiocruz Genomic Network [44]. Consensus sequences were assigned to viral lineages according to the nomenclature proposed by Rambaut [20] using the Pangolin software version 4.3 [45].

### 4.4. RNA Sequencing (RNA-Seq) Analysis

The quality of the 24 transcriptome libraries was checked using FASTQC [46], and the trimming was performed using Trimmomatic [47]. STAR tool [48] version 2.6 was applied to map the transcriptome libraries on the human genome (GRCH38.p12) from the ENSEMBL database [49] using the default parameters. The DESeq2 package [50] from R was used for differential gene expression analysis. Only the genes with an adjusted *p*-value < 0.01 and Log2 fold-change >|1.0| were considered for downstream analyses.

### 4.5. Enrichment Analysis

Gene Ontology (GO) was used for the enrichment of biological processes. The transcriptome analysis was performed using the R package GOstats [51]. The Benjamini and Hochberg (BH)-adjusted *p*-value method was applied for the enrichment with a cutoff of 0.01. The KEGG analysis was performed with the R/Bioconductor package pathview [52] using KEGG, Biomart, and org.hs.eg.db datasets. The enriched promoter motifs analysis was performed with ShinyGO 0.77 [53].

### 4.6. Interactome Analysis

To produce the interaction networks, we used the data from the BioGRID database https://thebiogrid.org/. Cytoscape software version 3.9.0 was used for the network analysis [54]. The twenty genes with higher values of betweenness centrality were kept, creating networks together with genes from different GO groups. Data about host proteins’ interaction with SARS-CoV-2 viral proteins were also added from the BioGRID COVID-19 Coronavirus Curation Project [55].

### 4.7. SARS-CoV-2 Single Nucleotide Polymorphism (SNP) Calling and Mutations of Interest Analysis

The reference genome of SARS-CoV-2 was obtained from the ENSEMBL database (genome assembly: ASM985889v3) [49]. The quality check and trimming of the SARS-CoV-2 sequencing libraries were performed using FASTQC [46] and Trim Galore [56], respectively. The libraries were mapped against the reference genome using Bowtie2 [57]. The SNP calling was performed with BCFtools, using the command mpileup [58]. The defining polymorphisms of Delta and Gamma were evaluated in our samples based on the Pango nomenclature [59].

## Figures and Tables

**Table 1:** Delta-enriched promoter motifs from DEGs.

Enriched Promoter Motif	TF	TF Family	FDR	Score
TAGGAAATCGAAAGT	IRF7 *	IRF	3.85 × 10^−5^	16
GGAAAGTGAAAGCAAA	IRF2	IRF	6.97 × 10^−5^	21
AAAGTGAAAGTGAAAGT	IRF1	IRF	0.000353	23
GAAAAGTGAAACC	IRF2	IRF	0.014020	14
GAAAGTGAAAGT	PRDM1	C2H2 ZF	0.024503	12

* Query gene. TF—transcription factor.

**Table 2:** Mutations of interest in the variants Delta and Gamma.

SARS-CoV-2 Protein	Delta SNPs	Gamma SNPs	Affected Host Protein	Delta Transcriptome Results	Gamma Transcriptome Results
Nsp3	I1091V, P2287S, A1306L, P2046L	S1188L, K1795Q	RIG-I/DDX58	Up	NDE *
Nsp6	-	ΔSGF	TBK1, IRF3, STAT1/2	NDE *	NDE *
Nsp12	T4992I	-	MDA5/IFIH1	Up	NDE *
Nsp13	H5401Y	-	STAT2	Up	NDE *
M	I82T	-	RIG-I/DDX58	Up	NDE *
ORF7a	V82A	-	BST2, STAT2	Up	NDE *
N	D63G, G215C, R203M, D377Y	P80R, R203K, G204R	RIG-I/DDX58, STAT2	Up	NDE *

* Not differentially expressed.

## Data Availability

The datasets used in study are available online via the Gene Expression Omnibus database under accession number PRJNA952972, and genome sequences were also deposited in GISAID (https://www.gisaid.org/ (accessed on 7 July 2023)), doi: 10.55876/gis8.230518zu (Supplementary Material S1).

## Supplementary Materials

The supporting information can be downloaded at: https://www.mdpi.com/article/10.3390/ijms241713146/s1.
