# Supplementary material for: Differential Type-I Interferon Response in Buffy Coat Transcriptome of Individuals Infected with SARS-CoV-2 Gamma and Delta Variants
Source: Int J Mol Sci. 2023 Aug 24;24(17):13146. doi: 10.3390/ijms241713146 (PMC10487928; doi:10.3390/ijms241713146)
Supplement: Supplementary file 1 [file ijms-24-13146-s001.zip › Supplementary captions.pdf]

SupplementaryFigure S1. Differentially expressed genes present in the KEGG SARS-CoV-2 pathway for Gamma (P1) and Delta transcriptomes. Genes in green are upregulated and in red are downregulated.

Supplementary Table 1. GO enriched terms for biological processes in Delta trancriptome.

Supplementary Table 2. GO enriched terms for biological processes in Gamma trancriptome.

Supplementary Table 3. Upregulated differentially expressed genes in Delta transcriptome.

Supplementary Table 4. Downregulated differentially expressed genes in Delta transcriptome.

Supplementary Table 5. Upregulated differentially expressed genes in Gamma transcriptome.

Supplementary Table 6. Downregulated differentially expressed genes in Gamma transcriptome.
