# Supplementary material for: Differential Type-I Interferon Response in Buffy Coat Transcriptome of Individuals Infected with SARS-CoV-2 Gamma and Delta Variants
Source: Int J Mol Sci. 2023 Aug 24;24(17):13146. doi: 10.3390/ijms241713146 (PMC10487928; doi:10.3390/ijms241713146)
Supplement: Supplementary file 1 [file ijms-24-13146-s001.zip › Supplementary material 1.pdf]

SUPPLEMENTAL TABLE

Data Availability

GISAID Identifier: EPI_SET_230518zu
doi: 10.55876/gis8.230518zu

All genome sequences and associated metadata in this dataset are published in GISAID's EpiCoV database. To view the contributors of each individual sequence with details such as accession number, Virus name, Collection date, Originating Lab and Submitting Lab and the list of Authors, visit 10.55876/gis8.230518zu

Data Snapshot

- EPI_SET_230518zu is composed of 18 individual genome sequences.
- The collection dates range from 2021-04-30 to 2021-09-02;
- Data were collected in 1 countries and territories;
- All sequences in this dataset are compared relative to hCoV-19/Wuhan/WIV04/2019 (WIV04), the official reference sequence employed by GISAID (EPI_ISL_402124). Learn more at https://gisaid.org/WIV04.
