# Supplementary figures and images for: Differential Type-I Interferon Response in Buffy Coat Transcriptome of Individuals Infected with SARS-CoV-2 Gamma and Delta Variants
Source: Int J Mol Sci. 2023 Aug 24;24(17):13146. doi: 10.3390/ijms241713146 (PMC10487928; doi:10.3390/ijms241713146)

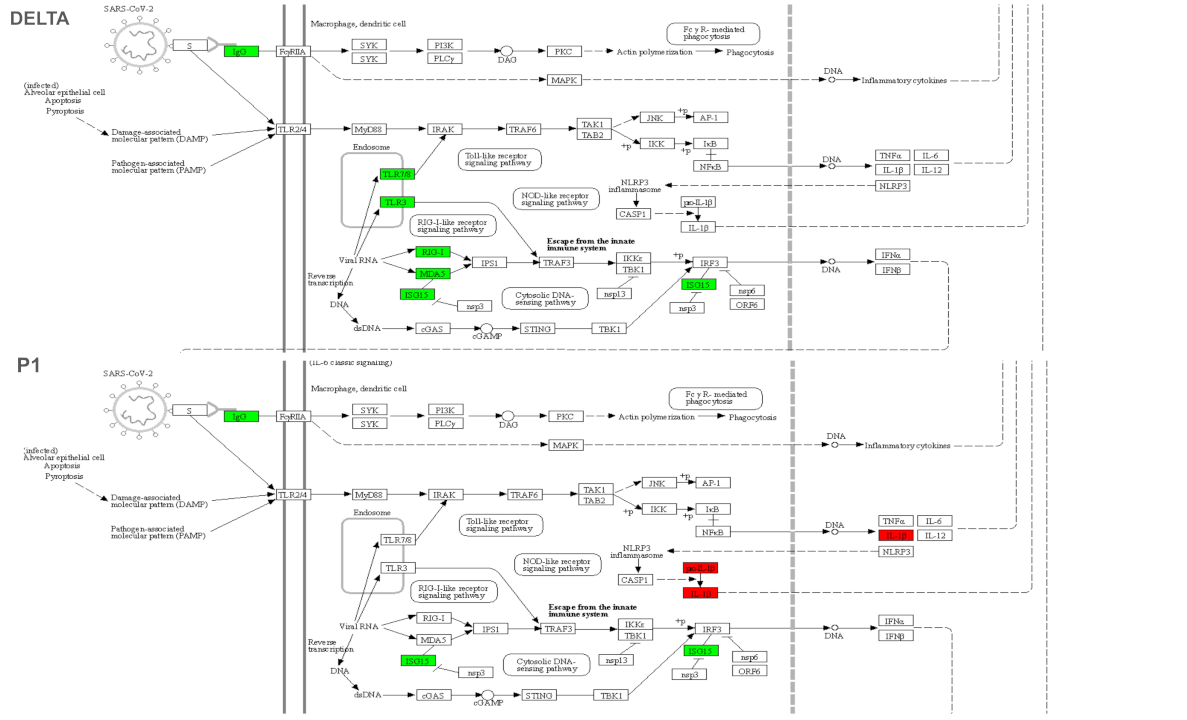

Supplement: Supplementary file 1 [file ijms-24-13146-s001.zip › Figure_S1.tiff]
